# The Role of Phagocytic Cells in the Achilles Tendon

**DOI:** 10.3390/ijms27052130

**Published:** 2026-02-25

**Authors:** Yasir Majeed, Maria Kokozidou, Clemens Gögele, Andreas Traweger, Christine Lehner, Herbert Tempfer, Gundula Gesine Schulze-Tanzil

**Affiliations:** 1 Institute of Anatomy and Cell Biology, Paracelsus Medical Private University, Prof.-Ernst-Nathan Straße 1, 90419 Nuremberg, Germany; yasir.majeed@pmu.ac.at (Y.M.); maria.kokozidou@pmu.ac.at (M.K.); clemens.goegele@pmu.ac.at (C.G.); 2 Spinal Cord Injury & Tissue Regeneration Center Salzburg, Institute of Tendon and Bone Regeneration, Paracelsus Medical University, 5020 Salzburg, Austria; andreas.traweger@pmu.ac.at (A.T.); christine.lehner@pmu.ac.at (C.L.); herbert.tempfer@pmu.ac.at (H.T.); 3 Austrian Cluster for Tissue Regeneration, 1200 Vienna, Austria

**Keywords:** macrophages, M1 and M2 polarization, tenophages, Achilles tendon, healing, heterotopic ossification, tendinopathy

## Abstract

Macrophages and other phagocytic cells are central regulators of tendon immunobiology, orchestrating inflammation, tissue repair, and extracellular matrix (ECM) remodeling in the tendons. They derive from circulating monocytes and resident tendon-specific populations, including tenophages. Macrophage polarization along the M1/M2 axis exerts a decisive influence on tendon healing trajectories. Activated M1 macrophages promote the early healing phase for debris clearance initiating the reparative cascade. However, their sustained activity leads to inflammation, ECM degradation, impaired healing, tendinopathy, and heterotopic ossification (HO). Conversely, a timed shift toward activated M2 macrophages promotes resolution of inflammation, angiogenesis, ECM deposition, and fibrocartilage formation, whereas excessive or prolonged M2 activity facilitates adhesion formation, fibrosis, scarring and HO. Recent single-cell and spatial profiling studies showed macrophage heterogeneity across tendon compartments, thereby extending the classical M1/M2 paradigm and underscoring the relevance of macrophages/resident tendon cell’s interaction in tendon-specific local niches. Mechanobiological stimuli (depending on magnitude, frequency and duration) further modulate macrophage phenotypes and tendon healing. Emerging coculture models and human tendon-on-chip systems provide high-resolution platforms for dissecting these spatiotemporal interactions. Promising therapeutic approaches comprise the application of extracellular vesicles, controlled mechanoloading regimens, and immunomodulatory biomaterials demonstrating potential to induce regenerative macrophage signatures for improved healing outcomes. Notably, platelet-rich plasma (PRP) formulations shape macrophage responses: leukocyte-rich PRP preferentially promotes M1 activity whereas leukocyte-poor PRP supports M2 polarization. Thus, mechano- and immunomodulatory strategies can offer precise control over macrophage dynamics. Regarding the Achilles tendon pathologies, such approaches are helpful by directing macrophage-mediated inflammation towards effective tendon healing outcomes.

## 1. Introduction

### 1.1. Tendon Cell Populations and Immune Cells

**Figure 1:**
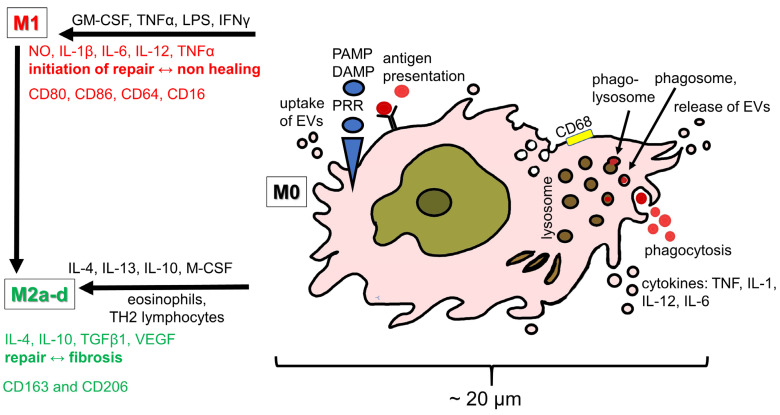
Typical characteristics of a classical macrophage. Classical macrophages display an irregularly shaped nucleus with a prominent nucleolus. A hallmark of their phagocytic function is the formation of phagolysosomes, generated by the fusion of internalized phagosomes with lysosomes. CD68 serves as a pan-macrophage marker and is expressed by non-polarized naïve M0 macrophages. Upon engagement of DAMPs or PAMPs via PRRs, M0 macrophages polarize toward a pro-inflammatory M1 phenotype. Alternatively, activated M2 macrophages comprise several subtypes (M2a–M2d), distinguished by their transcriptional signatures and mediator profiles [5]. DAMP: damage-associated molecular pattern (=alarmin), CD: cluster of differentiation, EV: extracellular vesicle, GM-CSF: granulocyte/monocytes colony stimulating factor, IFN: interferon, IL: interleukin, LPS: lipopolysaccharide, M-CSF: monocytes colony stimulating factor, NO: nitric oxide, PAMP: pathogen-associated molecular pattern, PRR: pattern recognition receptor, TGF: transforming growth factor, TNF: tumor necrosis factor, VEGF: vascular endothelial growth factor. This image was made with krita (version 4.4.7) by G. Schulze-Tanzil.

While several resident tendon cell populations including tenocytes, tenoblasts as tendon-specific fibroblasts, tendon-derived stem/progenitor cells (TSPCs) and endothelial cells are described, fibrochondrocytes are also reported in tendons occurring physiologically in selected areas (e.g., the enthesis or tendon parts exposed to pressure and shear forces due to twisted fiber bundles or gliding around a hypomochlion). However, fibrochondrocytes in the midportions of tendons are most often associated with degeneration where the tissue shows a so-called chondroid metaplasia underlining pathological conditions. Recent single-nucleus RNA sequencing has substantially expanded the understanding of tendon cellular heterogeneity, identifying up to 12 distinct cell subtypes, including an immune cell cluster in human hamstring tendon tissue [1]. Examination of immune cells within healthy tendon tissue, deriving from diverse human tendons and the rat Achilles tendon, consistently demonstrates low basal numbers of innate (e.g., phagocytic cells like resident macrophages) and adaptive immune cells (e.g., T cells) [2,3,4]. Among these immune cells in tendon, macrophages represent a key immune cell population with diverse homeostatic and reparative functions in tendons [5]. In tendon, macrophages are key actors during inflammation in tendinopathy and in healing tendons as reported in systematic reviews [6,7,8]. Non-immune and immune cells including human and murine T cells and macrophages interact intimately in tendon [2,9]. Tendon tissue homeostasis is regulated by macrophage-to-tenocyte ligand-receptor interaction: tenocytes release monocyte colony stimulating factor (M-CSF), which binds to the respective receptors on macrophages explaining the co-localization of human and mice macrophages and tenocytes [2,9]. Tendon stem cells also present as resident cells in the Achilles tendon and are known to exert immunomodulatory functions as shown in tendon healing where they communicate with macrophages [5,10,11]. Age-related changes further impact this interplay, as a progressive impairment of proliferation, clone formation and renewal of the TSPC cell pool has been observed in aging tendons; also, rat, murine and human macrophages show senescence, potentially contributing to impaired regenerative capacity [12,13,14]. From a morphological perspective, classical human macrophages appear as pale cells with a large, lightly stained nucleus containing a prominent nucleolus and often exhibiting an irregular nuclear contour. In contrast to monocytes, macrophages possess a broad and asymmetric rim of cytoplasm, notable for its heterogeneity due to abundant lysosomes and various cytoplasmic inclusions, containing carbon particles, cellular debris, and endocytotic vesicles. These cells exhibit a migratory phenotype, characterized by pseudopodia and lamellopodia and they typically express the pan-macrophage marker CD68 [15,16] (Figure 1). Additional characteristic markers detected in macrophages in a rat Achilles tendon model include receptors involved in phagocytosis and adhesion (e.g., CD11b), antigen presentation (e.g., human leukocyte antigen-DR locus [HLA-DR]), macrophage polarization (e.g., colony stimulation factor [CSF]-1R=CD115) and chemotaxis (e.g., C-C chemokine receptor type 2 [CCR2]) [17]. Highly specialized macrophage populations are present in nearly all tissues, including synoviocyte type A in the synovium of the joint capsules and tendon sheaths, osteoclasts in bone, Langerhans cells in the skin, Kupffer cells in the liver and alveolar macrophages in the lung [18]. The resident macrophages in different murine tendon types (limb and tail tendon) obviously differ [9].

### 1.2. Tendon Structure and Tissue Compartments in the Achilles Tendon

**Figure 2:**
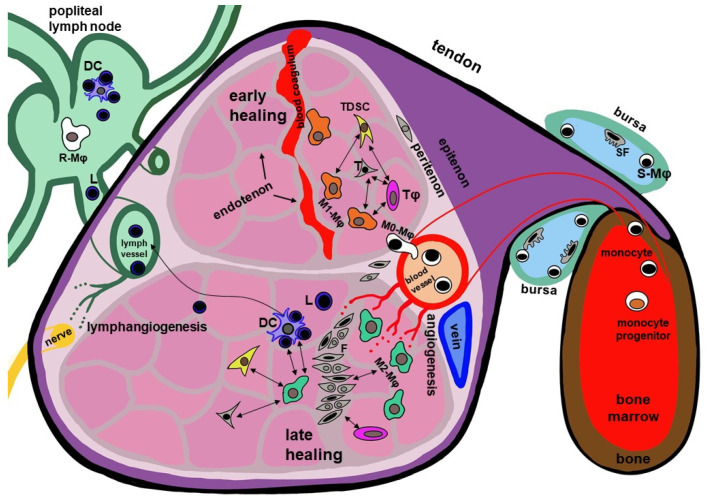
Macrophages (Mφ) in the Achilles tendon and their circulation dynamics. During early tendon healing, pro-inflammatory M1 macrophages accumulate at the injury site and release cytokines and chemokines that recruit additional leukocytes and orchestrate the initial immune response. As healing progresses (later healing, e.g., proliferation phase), a polarization shift toward M2 macrophages occurs. These anti-inflammatory and pro-regenerative macrophages display enhanced phagocytic capacity and promote ECM synthesis in tenocyte, angiogenesis, lymphangiogenesis and overall tissue repair. Achilles tendon-derived dendritic cells and lymphocytes can also enter lymphatic vessels within the epitenon and special tendon regions, after which they become detectable in the first draining regional lymph node (popliteal lymph node). Multiple interactions between macrophages and other tendon cell populations are established or presumed, including crosstalk with tenocytes, tendon-derived stem and progenitor cells (TDSCs, yellow triangle), dendritic cells and specialized tenophages. DC: dendritic cell (pale blue), L: lymphocytes (dark blue), T: tenocyte (gray, triangled), F: fibroblasts (gray, spindle-shaped), M0- (white)/M1-(orange)/M2-(turquoise)Mφ: M0-/M1-/M2 macrophages, R-Mφ: resident macrophage in lymph node, S-Mφ: synovial macrophages (synoviocyte type A), SF: synovial fibroblasts (synoviocyte type B), Tφ: tenophages (pink). The paratenon is not shown. This image was made with krita (version 4.4.7) by G. Schulze-Tanzil.

The hierarchical organization of the Achilles tendon, comprising distinct cellular populations and ECM niches, has been increasingly well characterized. Anatomically, the human Achilles tendon forms through the confluence of the tendons of the medial and lateral gastrocnemius muscle heads and the soleus muscle tendon [19]. Within its midsubstance, these fiber bundles exhibit a pronounced helical twist [20]. Clinically, two regions in the human Achilles tendon are particularly susceptible to injury: the twisted midportion which contains some fibrocartilage and the central zone of the enthesis, representing the tendon’s osseous attachment [21]. Throughout its length, the tendon is enveloped by the paratenon, a loose connective tissue layer that facilitates gliding against surrounding connective tissues and reduces friction [19]. Directly beneath the paratenon lies the epitenon, a thin connective tissue sheath covering the tendon’s surface. The epitenon and the next deeper connective tissue layer, the peritenon, contains an extensive network of blood and lymphatic vessels, and sensory and autonomic nerves. The peritenon surrounds each tendon fascicle, whereas the endotenon envelops the subfascicles and represents the innermost connective tissue layer. Together, these interfascicular collagenous connective tissue layers provide the tendon with its vascular, neural supply and lymphatic drainage thereby contributing to its compartmentalized ECM organization [22,23]. Thus, a distinction can be made between interfascicular (epi-/peri-/endotenon) and fascicular ECM (Figure 2).

Within tendon fascicles, resident macrophages are present as native cells positioned adjacent to the native tenocytes. Transcriptomic analyses in mice suggest active signaling crosstalk between these two populations [9]. Resident macrophages can be detected in tendons as early as embryonic day 15.5 in mice, with their numbers in contrast to tenocytes progressively increasing by proliferation during postnatal development [9]. Particularly, in flexor tendons, the tendosynovium serves as a major macrophage source, containing type A synoviocytes, a population of synovial macrophages. Although the human Achilles tendon lacks a tendosynovium, it is associated with two synovial bursae: the retrocalcaneal bursa, located between the ventral tendon site and the calcaneus, and a more superficial subcutaneous bursa situated dorsally [23,24]. Lymphatic vessels, predominantly localized in the epitenon and superficial tendon regions, play a critical role in leukocyte trafficking and thus, influence macrophage distribution and tendon immunobiology, particularly of the murine and human Achilles tendon [25,26] (Figure 2). Lymphangiogenesis is a central event in inflammatory responses, and vascular endothelial growth factor (VEGF-C), secreted by M2 macrophages, is essential for this process. Moreover, during the development of lymphatic vessels, mesodermal precursors of macrophages have been shown to contribute to the pool of lymphatic endothelial progenitor cells [26,27] and hence, might prospectively also be involved in lymphatic remodeling during tissue repair.

### 1.3. Immunobiology of the Achilles Tendon

**Figure 3:**
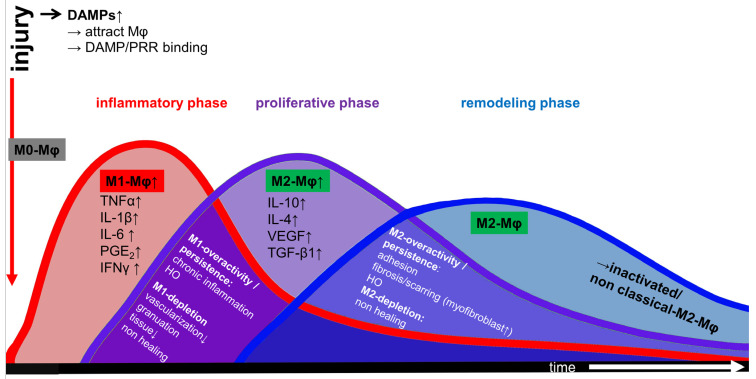
Macrophage polarization in the Achilles tendon healing phases. Tendon injury induces the release of damage-associated pattern (DAMPs) from disrupted tendon cells and extracellular matrix (ECM) fragments. Binding of DAMPs to pattern recognition receptors (PRRs) on naïve M0 macrophages drives M1 polarization. Pro-inflammatory M1 macrophages (M1-Mφ) dominate the early inflammatory phase and secrete mediators such as tumor necrosis factor α (TNFα) and Interleukin-1β (IL-1β), amplifying inflammatory cell recruitment and initiating extracellular matrix degradation. During the proliferative (also called: granulation) phase, macrophages progressively shift toward an M2 (M2-Mφ) phenotype. M2 macrophages release vascular endothelial growth factor (VEGF) and transforming growth factor β1 (TGF-β1), promoting angiogenesis, lymphangiogenesis and ECM synthesis, including collagen type 3 deposition. Excessive or prolonged M2 activity may lead to pathological recruitment and differentiation of resident tendon-derived stem/progenitor cells into myofibroblasts, contributing to fibrosis and adhesion formation. In the late remodeling phase, macrophage numbers drop and M2 macrophages become inactive. The red arrow marks the time point of injury. The white arrow (X-axis) marks the proceeding time. This image was made with krita (version 4.4.7) by G. Schulze-Tanzil. The red arrow marks the time point of injury. The white arrow (X-axis) marks the time after injury.

Both the human and rat Achilles tendons contain dynamic immunological niches in which resident stromal cells and infiltrating immune cells derived from the circulation cooperate to restore and maintain tissue homeostasis and respond to injury. This bidirectional crosstalk—between circulating immune cells, locally resident macrophages, and tendon-resident stromal cell populations—has emerged as a defining feature of tendon immunobiology [28,29]. Increasing attention has focused on how macrophage polarization influences the development of murine Achilles tendon disorders and shapes the healing trajectory following injury or clinical interventions [30]. Consequently, therapeutic approaches that modulate macrophage phenotype, particularly those aiming to enhance anti-inflammatory or pro-regenerative M2-like responses, are being actively explored in several experimental models like the rat and murine Achilles tendon model and the murine rotator cuff model [30,31,32]. Following tendon injury, macrophages orchestrate the inflammatory and reparative healing processes through dynamic polarization states, broadly characterized as M1 and M2 phenotypes in an acute rat Achilles tendon tenotomy model [17]. Tendon healing progresses through three partially overlapping phases: the inflammatory, proliferative and remodeling phase [33] (Figure 3). The initial inflammatory phase of Achilles tendon healing is marked by rapid infiltration of neutrophils followed by monocytes and macrophages, which release pro-inflammatory mediators that promote debris clearance and activate resident stromal cells in human rotator cuff tear patients but also in animal tendon injury models [7,34,35]. As inflammation subsides, the proliferative phase is characterized by increased production of growth factors—including VEGF and transforming growth factor beta (TGF-β)—which promote angiogenesis, fibroblast recruitment, and granulation tissue formation in a streptozotocin (STZ)-induced murine diabetic wound healing model [36]. The final remodeling phase involves reorganizing and maturation of collagen fibers, gradually restoring tensile strength and functional load-bearing capacity [37]. Macrophage polarization is regulated by distinct cytokine milieus. M1 macrophages, induced by stimuli such as lipopolysaccharide (LPS), interferon-gamma (IFN-γ), or tumor necrosis factor-alpha (TNF-α), exhibit pro-inflammatory and phagocytic activities, essential for pathogen defense and early injury clearance [38]. In contrast, M2 macrophages generated in response to cytokines such as interleukin-4 (IL-4), IL-10 or IL-13, support the resolution of inflammation, ECM deposition, and tissue regeneration [18,39] (Figure 1). Although the classical M1/M2 paradigm remains widely used, recent findings suggest a more nuanced spectrum of macrophage states within tendon tissue in both humans and animal models (Figure 1) [40,41,42]. Importantly, tendons harbor their own resident macrophage population, identified in cultured primary rat Achilles tenocytes and murine Achilles and human semitendinosus tendons [43]. Macrophages contribute to innate immune surveillance through the expression of pattern recognition receptors (PRRs), enabling the recognition of pathogen-associated molecular patterns (PAMPs) present on microbes, and damage-associated molecular patterns (DAMPs) [44,45]. DAMPs—also known as “alarmins”—are released from stressed, injured, or necrotic cells [46]. In the context of human supraspinatus tendinopathy in rotator cuff tears and rat Achilles tendon injury, the damaged tenocytes are a key source of alarmins such as S100A8 and S100A9 [34,47,48]. As it has been observed in several human tendons while studying tendinopathy, their interaction with resident or infiltrating macrophages triggers macrophage’s cytokine release, vascular activation, and further immune cell recruitment, amplifying the early inflammatory response [45,49]. Collectively, these processes illustrate how tightly coordinated immune-cell/tenocyte interactions shape the inflammatory and reparative landscape of the Achilles tendon. Understanding these mechanisms is essential for designing targeted immunomodulatory therapies aiming to optimize tendon repair and preventing chronic degeneration.

### 1.4. Monocytes–Macrophages–Dendritic Cell Axis in Tendon

Macrophages in tendon arise from two distinct ontogenetic pathways. In mammals including humans, circulating monocytes originating from the bone marrow migrate via the blood stream into tendon, where they differentiate into macrophages and participate in immune surveillance and tissue repair. In parallel, another population of tissue-resident macrophages derives directly from primitive yolk-sac macrophages during the embryonic development [50]. Within tendon, macrophages are not only phagocytic but also acquire the capacity for antigen presentation, enabling interactions with other immune cells. This could be explained by the fact that macrophages and dendritic cells (DC) derive from a common macrophage-DC-restricted precursor cell [51]. However, being distinctly different cell types, DCs and macrophages present some functional overlap by sharing antigen presenting capacity and cytoplasmic protrusions for cell interaction [51,52]. Recent studies have highlighted intimate communication between human tendon-resident cells originating from the semitendinosus muscle or the Achilles tendons and DCs, as well as MSCs interacting with macrophages in a murine Achilles tendon model suggesting a coordinated immunoregulatory network within the tendon [53,54]. Macrophages constitute a heterogeneous population with diverse functional phenotypes. The pan-macrophage marker CD68 is expressed on M0, M1 and M2 macrophages. Classical pro-inflammatory M1 macrophages are characterized by the expression of CD80, CD86, CD64, and CD16, whereas anti-inflammatory, tissue-reparative M2 macrophages typically express CD163 and CD206; the latter is a surface lectin serving as a mannose receptor as seen in patients after rotator cuff repair [55]. In a rat Achilles tendon model, mixed or intermediate macrophage phenotypes are also well documented, reflecting the spectrum of functional states that macrophages can adopt in vivo [17]. In addition, a resident macrophage-like population has also been described in both rat and murine Achilles and human semitendinosus tendons [43]. Macrophages play a central role in tendon repair, including both tendon-to-tendon and tendon-to-bone healing, and are key mediators of tendinopathy pathogenesis [56,57]. The functional activity of macrophages is further modulated by exosomes, including stem-cell and DC-derived exosomes, which can direct macrophage polarization and activity [53,58]. M1 and M2 macrophages determine the inflammatory niche in tendon lesions and contribute to modulating the phenotype of stem cells (chondrogenic or osteogenic) and their activation [59]. This bidirectional communication underscores the critical role of the monocyte–macrophage–DC axis in both tendon homeostasis and repair, highlighting macrophages as central orchestrators of tendon immunobiology.

## 2. Relevant Sections

### 2.1. Specialized Tissue-Specific Macrophages in Tendon: Tenophages

Tendons, like many other tissues, harbor specialized resident macrophage populations that contribute to local immune surveillance and tissue homeostasis. Analogous examples include synovial macrophages in the tenosynovium and bursae, Kupffer cells in the liver, and Langerhans cells in the skin; additional organs such as the spleen contain multiple macrophage subsets adapted to distinct microenvironmental tasks [60]. In line with this paradigm, a unique population of resident phagocytic cells with macrophage-like properties has recently been identified in murine Achilles tendons and in human semitendinosus muscle tendons. These cells, termed tenophages, occupy the tendon core and appear to represent a tissue-specialized macrophage lineage.

Tenophages express CX3R1, the receptor for the chemokine fractalkine/CX3CL1, a signaling axis classically associated with macrophages chemotaxis, tissue residency, and inflammation responses. Functional experiments demonstrate that primary rat Achilles tendon cell migration was attenuated upon CX3CR1 inhibition, underscoring the importance of fractalkine signaling in their behavior and potentially in their spatial organization within tendon tissue [43]. In human semitendinosus muscle tendon sections, these cells were also observed within the walls of blood vessels, suggesting interactions with vascular signaling niches and possible roles in immune cell trafficking [43].

Notably, tenophages co-express CX3CL1, CX3CR1, and epiregulin (EREG), molecules associated with cell proliferation, angiogenesis, and fibrotic remodeling during inflammatory responses. These processes are central to the pathogenesis of tendinopathy, implicating the CX3CL1/CX3CR1/EREG axis as a potential driver of pathological ECM turnover and aberrant tissue repair. Consequently, modulation of this signaling pathway may represent an appealing therapeutic strategy for tendon disorders characterized by chronic inflammation, disorganized healing, and fibrosis [43].

Together, the identification of tenophages expands the conceptual framework of tendon immunobiology, highlighting that tendons, despite their relatively sparse cellularity and immune-privileged characteristics, maintain a resident myeloid cell population with distinctive signaling features and potential relevance to both physiological remodeling and disease progression.

### 2.2. Role of Macrophages in Achilles Tendon Homeostasis and Repair

In uninjured tendons, macrophages maintain tissue homeostasis through continuous surveillance, debris clearance, and immunoregulation.

Both monocyte-derived and embryonically established macrophages contribute to homeostasis. Their trafficking and drainage depend on the lymphatic vessel network. In the human Achilles tendon, lymphatics are concentrated within the epitenon and superficial tendon layers and drain toward the popliteal lymph nodes, enabling antigen transport and immune cell recirculation, e.g., of lymphocytes and DCs [25] (Figure 2).

Through these combined surveillance, clearance, and immune-modulating functions, macrophages act as key guardians of tendon homeostasis, preserving tissue stability under physiological loading conditions.

Macrophages are central regulators of tendon healing including the Achilles tendon in a rat model, orchestrating both the early inflammatory response and the subsequent reparative and remodeling phases [61,62]. Following rat Achilles tendon injury, they were rapidly recruited to the damaged site [63], where they release pro-inflammatory mediators such as IL-1β, TNF-α, and prostaglandin E2 (PGE_2_). In humans and in murine models, these cytokines initiate tendon ECM degradation, an essential early step of debris removal, but they may also precipitate tendinopathic changes when dysregulated [64,65,66].

Dynamic macrophage polarization into M1 or M2 subsets governs the balance between inflammation and regeneration. M1 macrophages amplify inflammation, whereas M2 macrophages promote resolution through IL-10 and IL-4 signaling and support tissue remodeling [67,68]. Crosstalk between tenocytes and macrophages significantly influences polarization and progression of early healing as shown in the equine digital flexor tendon and human supraspinatus tendon cell models [69,70] (Figure 3). In vivo, a high M1/M2 macrophage ratio is characteristic of early tendon repair as shown at day 3 in a rat Achilles tendon transection model followed by T helper and regulatory T (Treg) cells [29]. Consistent with this, markers of M1 macrophages (CCR7, CD11b, IL-1β, and IL-6) are elevated shortly after Achilles tenotomy and repair, while M2 markers (CD163 and IL-10) rise during late healing around day 28 [17] (Figure 2). The pro-regenerative function of M2 macrophages in tendon healing is well recognized based on results gained in several animal models [71], with their dominance promoting the formation of regenerative tissue [11]. During the late remodeling phase, macrophage numbers drop and their phenotype shifts to an “inactivated” or non-classical M2 state (Figure 3) [72]. Accordingly, therapies such as PRP and activated autologous serum have been suggested to improve rotator cuff healing as it induces M2 macrophage polarization in vitro [55]. Another study reported that macrophages treated with stem cell-derived extracellular vesicles (EVs) undergo M2-like polarization in a murine Achilles tendon rupture model, which can potentially be exploited in future therapeutic approaches [11]. A more recent work suggested that light emitting diode (LED) treatment improved Achilles tendon healing in the murine model by shifting macrophages toward the M2 phenotype [73].

Both depletion and overactivity of macrophages subsets impair tendon repair. Depletion of M1 macrophages compromises healing in the early phases and leads to poor healing and reduced vascularization and granulation tissue formation as shown in other tissues injuries such as liver and heart [74,75], whereas excessive M1 activation drives chronic inflammation [76]. Conversely, M2 depletion delays wound closure, but excessive M2 activity leads to increased collagen deposition and fibrosis, as shown in another model of lung fibrosis [77,78]. These effects largely reflect the role of M2 macrophages as a major source of TGF-β1, which stimulates ECM synthesis in a human Tendon-on-a-Chip (hToC) model [79], recruits progenitor cells in both human and murine models and induces their differentiation into myofibroblasts [80]. While essential for normal repair, excessive TGF-β1 signaling can drive adhesion formation and fibrosis, both adverse outcomes of tendon healing [81]. Along these lines, macrophage-derived miRNAs have also been linked to adhesion development in a murine flexor digitorum longus tendon model [82]. Recent work has identified a pro-fibrotic macrophage phenotype in tendon fibrosis, which is characterized, next to TGF-β1 secretion, by the expression and secretion of osteopontin/phosphoprotein 1 in several murine tendons such as Achilles, supraspinatus and flexor digitorum tendons [81]. In contrast, a “folate receptor beta (FOLR2^+^)” macrophage subtype has been proposed to exert anti-fibrotic effects in human and murine tendon subjects [83]. These insights point toward precision strategies targeting TGF-β1 signaling and macrophage plasticity to prevent post-injury adhesions [81].

Beyond fibrosis, aberrant macrophage polarization also contributes to heterotopic ossification (HO), the pathological formation of bone within healing or tendinopathic tendons, as seen in a murine Achilles tendon model [84]. Macrophages were strongly implicated in the inflammatory microenvironment that initiates HO, representing another hallmark of aberrant repair observed after acute Achilles tendon injury and chronic tendinopathy in rat and murine models [85,86]. In a rat model of Achilles tenotomy-induced HO, macrophages persist throughout the endochondral ossification process, residing within and around the evolving ectopic bone and bone marrow-like tissue, and neurotrophines (NT) secreted by macrophages seem to play a major role in HO formation [85]. A key mechanistic insight is that macrophages serve as a principal source of the osteogenic factor NT-3. Immunolabeling shows NT-3 co-localization with both M1 and M2 macrophages at the injury site [87]. This paracrine function is enhanced by inflammatory stimuli: in vitro studies using the murine RAW264.7 macrophage cell line demonstrated a significant increase in NT-3 secretion following LPS stimulation [88,89]. The foundational importance of macrophages in vivo is demonstrated in conditional-on knock-in mice by depletion experiments, where systemic elimination of macrophages using clodronate-loaded liposomes results in a profound reduction in both HO volume and the local expression and systemic concentration of NT-3 [88,90]. Thus, macrophages not only coordinate immune and reparative phases but also act as central drivers of ectopic bone formation, integrating inflammatory cues with osteogenic signaling in the HO pathway.

### 2.3. Macrophage Polarization: A Therapeutic Target in Achilles Tendinopathy

Tendinopathy is widely regarded as a chronic, multifactorial disorder arising from prolonged mechanical overloading, repetitive microtrauma and inadequate repair, ultimately resulting in degenerative changes within the tendon ECM. Mechanical overuse increases the metabolic and oxygen demand of tenocytes. In the Achilles tendon, a region of relative hypovascularity located 2–6 cm proximal to the calcaneal insertion, is particularly susceptible to hypoxia during overloading [20]. The resultant low-oxygen microenvironment predisposes tenocytes to metabolic stress and promotes a sustained inflammatory response, reflected in elevated local levels of PGE_2_, IL-1β, and TNF-α [6]. Age-related alterations, systemic inflammatory conditions, and metabolic disorders such as diabetes mellitus further increase susceptibility to tendinopathic degeneration, particularly of the Achilles tendon models as shown by the systematic review and meta-analysis of De Luca et al. [91,92,93].

Macrophage accumulation is a consistent feature in tendinopathy in humans, and their numbers are significantly increased in chronic Achilles tendinopathy compared with healthy tendon tissue [94,95,96]. In human biopsy studies, macrophages were detected in 96% of tendinopathic Achilles tendon samples, with markedly higher density than in non-pathological controls, suggesting a central role for macrophage-driven inflammatory dysregulation in disease progression [95].

Pro-inflammatory M1 macrophages contribute to a pathogenic inflammatory milieu by releasing TNF-α, IFN-γ, IL-1β, and activation of inducible nitric oxide synthase [97], as shown for the human Achilles tendon [56]. These mediators activate canonical downstream signaling pathways, including Nuclear factor (NF)-κB and NLR family pyrin domain containing 3 inflammasome (NLRP3) pathway, both of which are activated by DAMP and PAMP signals and induce the transcription of inflammatory genes, e.g., of pro-inflammatory cytokines, in both human and murine rotator cuff and human biceps tendons [6,98,99]. Sustained M1 dominance may also be promoted by impaired Janus kinase/signal transducer and activator of transcription (JAK2/STAT3) signaling. This pathway normally mediates the macrophage shift to the M2 polarization. In tendinopathy, macrophages exhibit elevated expression of the protein tyrosine phosphatase 1B (PTP1B), a negative regulator of JAK2 activation, thereby disrupting mitochondrial homeostasis and suppressing the M1 to M2 phenotypic shift essential for the resolution of inflammation in human and murine Achilles tendinopathy models [100]. In contrast, M2 macrophages exert anti-inflammatory and reparative influences by producing IL-1 receptor antagonist, IL-10, IL-13, arginase 1, and matrix metalloproteinases (MMPs), and growth factors such as VEGF, insulin-like growth factor 1 (IGF1), TGF-β1 and platelet-derived growth factor (PDGF), factors that promote ECM remodeling, angiogenesis, and wound-healing [101,102,103]. Insufficient M2 activation or an unfavorable M1/M2 ratio therefore contributes directly to failed resolution and pathological remodeling.

The therapeutic modulation of macrophage phenotypes is an emerging strategy to restore tendon homeostasis. Interestingly, a recent study demonstrated that a tendon-derived ECM hydrogel, particularly when combined with tendon-derived stem cell (TDSC) exosomes, promotes M2 macrophage polarization, suppresses inflammation, and enhances tenogenic differentiation in vitro [104]. In vivo, this biomaterial therapy improved tendon histological organization, increased expression of tenogenic markers, augmented biomechanical strength, and shifted macrophage populations toward a regenerative M2 phenotype in a tendinopathy model [104]. Such findings highlight the therapeutic potential of macrophage-centered interventions to rebalance the inflammatory niche and mitigate tendinopathic degeneration.

### 2.4. Role of Macrophages in Tendon-to-Bone Healing

The tendon-to-bone interface forms the mechanical and biological junction between tendon and bone. Histologically, the enthesis comprises four zonal layers: tendon, uncalcified fibrocartilage, calcified fibrocartilage, and bone [105]. Because tendon and bone differ substantially in composition and mechanical behavior—exhibiting elastic moduli of ≈100 MPa (tendons) and ≈20 GPa (bones), respectively—this pronounced mismatch in tissue composition and mechanical properties renders the enthesis a potential weak point when subjected to elevated mechanical loads as it has been observed in immature bovine anterior cruciate ligament (ACL) samples [106]. This is exemplified in the Achilles tendon, the most heavily loaded tendon in the human body, which can experience forces exceeding ten times body weight during dynamic activity [107].

### 2.5. Early Injury Phase: M0 Recruitment and M1 Polarization

Following injury, macrophages originating from the circulation, bone marrow, and—depending on tendon type—from the synovial environment infiltrate the interface and rapidly polarize toward a pro-inflammatory M1 phenotype [108]. M1 macrophages secrete IL-1β, TNF-α, and IL-6 cytokines required for debris clearance but are also capable of impairing repair and are associated with tendinopathy when produced excessively. IL-1β and TNF-α enhance ECM degradation through upregulation of MMPs and NF-κB activation in tenocytes and tendons [64,98,109]. Moreover, sustained M1 activity promotes osteoclastogenesis and bone resorption at the enthesis, further compromising biomechanical integrity [110,111,112]. Several factors skew macrophages towards this M1 profile; for instance, leptin exacerbates M1 polarization in a rat rotator cuff repair model [113] and LR-PRP induces M1 responses in a patellar tendon injury murine model [114].

### 2.6. Transition Toward Repair: M2 Polarization and Pro-Regenerative Activity

M2 macrophages, characterized by the expression of markers such as CD206 and arginase (Arg-1), become predominant at the tendon-to-bone interface during the repair and remodeling phases, following the initial inflammatory stage dominated by M1 macrophages in rat and murine models [115,116]. The accumulation of M2 macrophages is thought to arise both from the repolarization of M1 macrophages and the activation of circulating M0 macrophages under stimuli such as anti-inflammatory cytokines, IL-4 and IL-13 [113,117]. M2 macrophages secrete anti-inflammatory cytokines, including IL-4 and IL-10, which contribute to an environment favorable for tissue regeneration [103,116]. M2 macrophages further promote vascularization and osteogenesis by producing TGF-β1, VEGF, and BMP-2, which facilitate fibrocartilage formation, angiogenesis and osteogenesis [118,119,120]. TGF-β1 expression peaks at around day 10 post-injury and is strongly associated with fibrocartilaginous ECM deposition; however, excessive or prolonged TGF-β1 signaling promotes fibrotic scar formation characterized by type 3 collagen accumulation, limiting optimal enthesis regeneration [121]. Importantly, recent studies highlight that mechanical loading shapes macrophage phenotype. Particularly moderate exercise induces a switch toward M2 macrophages via the JAK/STAT pathway, leading to enhanced TGF-β1 secretion by macrophages, which in turn stimulates healing in a mouse wound healing model, and MSC chondrogenesis and glycosaminoglycan deposition in murine rotator cuff tendon-to-bone healing models [32,97,122,123]. Neutralization of TGF-β1 in macrophage-conditioned media significantly reduced glycosaminoglycan deposition and the expression of chondrogenic markers (collagen type 2 alpha 1 chain, SOX9, and aggrecan) in MSCs, confirming its central role [32]. In vivo, treadmill-induced mechanical loading in a murine rotator cuff model increased TGF-β1 expression at the tendon-to-bone interface, resulting in enhanced fibrocartilage formation and biomechanical strength [32]. Collectively, M2 macrophages mediate a critical shift from inflammation to regeneration, yet their secretome includes mediators with both beneficial and detrimental potential, underscoring the need for precise spatiotemporal regulation of macrophage phenotype during tendon-to-bone healing.

### 2.7. Therapeutic Modulation of Macrophage Phenotypes

Because macrophage polarization strongly influences enthesis regeneration, multiple therapeutic strategies aim to modulate macrophage phenotypes.

#### 2.7.1. Targeting M1 Activity

Reducing M1-driven inflammation enhances enthesis healing quality. TNF-α inhibition decreases M1 macrophage numbers and improves fibrocartilage restoration in a tendon-to-bone repair murine rotator cuff model [124]. Thus, controlling pro-inflammatory overactivity results in improved regeneration of the fibrocartilage layer [124]. Similarly, IL-1β blockade or downstream targeting of NF-κB signaling such as inhibition via IκB kinase (IKK), which activates the NF-κB pathway, improves outcomes in a murine model [98].

#### 2.7.2. Promoting M2 Responses

Bone marrow derived MSC exosomes enhance tendon-to-bone healing by promoting M2 polarization, increasing VEGF expression, and suppressing M1-driven pro-inflammatory cytokines in a rotator cuff reconstruction rat model [125]. M2 macrophages produce TGF-β, whose isoforms play a significant role in scar formation and healing [126,127]. In a rat model, the use of exogenous TGF-β1 in order to promote tendon-to-bone healing after an acute rotator cuff injury resulted in increased biomechanical strength at the wound site, nevertheless, at the cost of excessive scar tissue formation [128]. TGF-β3 is another isoform of TGF-β normally scarce in tendon-to-bone healing. It promotes fibrocartilage formation [121]. The latter is supported by another study where the use of exogenous TGF-β3 in a rat model supports zonal fibrocartilage regeneration and yields improved structural and mechanical outcomes with reduced fibrosis [129].

Overall, targeted macrophage modulation represents a promising strategy to achieve biological fixation and functional regeneration of the tendon-to-bone interface. Future approaches will likely combine biomaterials, mechanotherapy, and immune engineering to optimize macrophage dynamics during the sequential phases of enthesis healing.

### 2.8. Coculture and Microphysiological Models of Tendon–Macrophage Interactions

Conventional coculture systems have substantially advanced our understanding of how macrophages regulate tendon homeostasis, inflammation, fibrotic remodeling, and the factors influencing macrophage polarization [130]. Early work employing indirect coculture of Achilles tenocytes with peripheral blood mononuclear cells demonstrated robust induction of tenocyte inflammatory responses, including increased expression of IL-1β, TNFα, IL-6 and MMP1 [131]. Similarly, coculture of macrophages with cytokine-activated supraspinatus tenocytes enhanced the secretion of pro-inflammatory cytokines (IL-6 and IL-8) and monocyte chemoattractant protein-1 (MCP-1) [70]. These findings highlight that even without direct cell–cell contact, macrophage-derived factors profoundly modulate tenocyte inflammatory signaling.

In addition to pro-inflammatory interactions, several studies have explored macrophage polarization within tendon-specific microenvironments. Exposure of rotator cuff tendon tissue and bursa explants to PRP or autologous activated serum promoted M2-type macrophage polarization [55]. Direct and indirect cocultures of an equine cell model (digital flexor tenocyte and macrophages) using macrophages of defined phenotypes revealed that M1 macrophages drive ECM-degrading activity in cocultures (e.g., MMP induction) whereas M2 macrophages promoted tissue inhibitor of matrix metalloproteinases (TIMP-1) release without requiring direct cell contact. Induced MMP gene expression was also observed in tenocytes in indirect cocultures [130]. Cocultures of tenocytes (derived from human healthy semitendinosus muscle and tendinopathic Achilles tendons) with monocyte-derived DCs, the latter treated with oxidized phospholipids, further demonstrated a strong stimulatory influence of this immune cell population particularly on diseased Achilles tenocyte proliferation [54]. More complex multicellular models have examined macrophage interactions with alternative therapeutic cell candidates. Innis et al. reported that while tenocytes exerted minimal inflammatory influence on adipose tissue-derived stem cells (ASCs), M1 macrophages potently induced pro-inflammatory signaling in ASCs, an important consideration for stem cell-based tendon therapies [132]. Additional coculture studies evaluating therapeutic biomaterials showed that high-molecular-weight hyaluronan or collagen suppressed CD14 expression on M1 macrophages, while enhancing collagen type 1 and CD44 in Achilles tenocytes [133].

#### 2.8.1. Tendon-on-Chip Models Incorporating Macrophages

Microphysiological hToC platforms now allow recreation of spatiotemporally dynamic tendon immune microenvironments. Early hToC systems focused on tenocyte–T cell interactions within compartmentalized human patellar tendon models [134,135].

Ajalik et al. (2025) developed an hToC platform integrating human tendon-derived cells, and physiologically relevant hydrogel matrices in order to study peritendinous adhesion formation [79]. This model allowed direct analysis of macrophage-driven tenocyte-myofibroblast transdifferentiation, revealing that resident tendon macrophages, without exogenous TGF-β1, can potentiate ECM remodeling. Incorporation of a vascular compartment composed of endothelial cells and circulating monocytes further replicated essential steps of tendon fibrosis: endothelial activation, monocyte transmigration, cytokine release and tissue-contractive myofibroblast formation, mediated in part through mTOR activation [79,136]. This interesting and complex model replicated crucial steps observed in clinical tendon fibrosis including vascular inflammation, cytokine release, monocyte endothelial transmigration and myofibroblast formation with tissue contraction implicating the activated mTOR signaling pathway [79].

These studies also underscored the central role of monocyte chemoattractant protein-1 (MCP1) in orchestrating immune–stromal cell crosstalk. In tendon–endothelial cocultures, MCP1 secretion was markedly upregulated [79]. MCP1 is mainly produced by epithelial cells, endothelial cells, monocytes/macrophages, fibroblasts, astrocytes and microglial cells [137,138,139]. These findings dovetail with established roles of MCP1 as a key driver of monocyte, microglia and memory T cell recruitment during tissue injury [79,140]. This also indicated that the tissue-resident macrophages play a role in recruiting circulating macrophages.

Similarly, coculturing tendon cells with macrophages without adding exogenous TGF-β1 showed that native macrophages can potentiate the fibrotic activity by themselves, thus indicating the native macrophage–tenocyte crosstalk resulting in pathological ECM remodeling [79]. Moreover, exogenous TGF-β1 significantly accelerates the fibrogenic response in tendon via myofibroblast differentiation, resulting in increased collagen type 1 and 3 and collagen type 3/1 ratio, heat shock protein (HSP)47, and alpha smooth muscle actin (α-SMA) expression in tendon cells [79].

#### 2.8.2. Other Complex Multicompartmental Models

Additional engineered models have provided insight into the relative contributions of intrinsic and extrinsic macrophage populations. A core tendon–peritenon system demonstrated that bone marrow-derived extrinsic macrophages reduce tissue breakdown mediated by intrinsic tendon cells during healing [141]. Importantly, accurate reproduction of fluid-flow dynamics, known to strongly influence monocyte–endothelial interactions, is essential for faithful replication by such models [142]. Enthesis-on-chip and myotendinous models have recently been developed to study region-specific biomechanics and inflammation, although macrophages have not yet been incorporated into these systems [143,144]. Together, these conventional coculture and next generation microphysiological models provide complementary mechanistic insights, establishing macrophages as central regulators of tendon inflammation, repair, fibrosis, and immune–stromal signaling. They also present powerful platforms for testing biomaterials, cell therapies, and immunomodulatory strategies in precisely controlled tendon microenvironments.

### 2.9. Effectors Influencing Macrophage Polarization in Tendon Healing and Tendinopathy

Typical polarization-dependent differences between M1 and M2 macrophages and the factors influencing their phenotypic shift are summarized in Table 1.

The effects of mechanotherapy/physiotherapy on macrophages have also been demonstrated on 3D tendon-constructs with primary tenocytes isolated from human M. semitendinosus and gracilis tendons [147]. A more recent study evaluated the effect of mechanical force on macrophage phenotypes deriving from appropriate stimulation of the human pro-monocytic cell line U937 in a 3D matrix model [145]. Pro-inflammatory M1 macrophages were found to be more responsive to low mechanical strain (3%), showing significant c-Fos upregulation and increased expression of TNF-α, IL-1β, and MMP3, whereas moderate strain (6%) promoted a shift toward an anti-inflammatory phenotype with elevated IL-10 and CD163 expression. However, M0 macrophages required a higher mechanical strain (12%) for the phenotypic shift into either pro-inflammatory or anti-inflammatory phenotypes, and with the increasing strain, they tend to shift more towards the M2 type [145]. Thus, the mechanical environment plays an important role in macrophage polarization, offering a mechanistic basis for the beneficial effects of controlled mechanical loading in tendinopathy rehabilitation. Ultrasound-based therapies have also been explored, but so far not reported for the Achilles tendon. They have shown promising results in a rat model of cartilage repair, in neural tissue and they supported the repair of bone fractures by stimulating osteogenesis [148,149,150,151]. The effect of low-intensity pulsed ultrasound (LIPUS) in tendon-to-bone healing has been thoroughly studied in a rat model [146], where they found out that the therapeutic efficacy of LIPUS is critically dependent on macrophage activity. When macrophages were depleted, the beneficial biomechanical effects of LIPUS disappeared. Early LIPUS exposure after injury enhanced CCR7^+^ M1 macrophage recruitment and inflammation, whereas treatment at later stages promoted CD163^+^/IL-10^+^ M2 polarization and tissue restoration. These findings confirm that LIPUS exerts its regenerative effects by modulating macrophage polarization dynamics, underscoring the importance of timing and strain amplitude in mechanotherapy-based tendon healing [146].

PRP therapy has also been explored as one of the potential targeted therapies in attenuating macrophage polarization and tendon healing [114]. Nishio and colleagues demonstrated the use of two types of PRP, i.e., leukocyte-rich (LR-PRP) and leukocyte-poor (LP-PRP) in an in vivo mouse patellar tendon model [114]. LR-PRP led to increased macrophage polarization to M1 type and expedited the initiation of the inflammatory phase of healing, and the LP-PRP enhanced the polarization to M2 type, aiding in the reparative phase [114] (Table 1). This indicated the potential of LP-PRP to be used as a targeted anti-inflammatory therapy. It seems that LR-PRP is beneficial for the early phase of tendon healing and a major effect of it is that it promotes type 1 collagen remodeling and angiogenesis [152,153]. Inflamed tenocytes from the semitendinosus muscle tendon presented reduced pro-inflammatory cytokines (IL-6) when exposed to PRP [154]. Additionally, PRP, demonstrated in diverse in vivo settings of tendon and ligament tears including the Achilles tendon, increased the tenocyte/ligamentocyte metabolic response, ECM expression, angiogenesis and healing, as reported in a systematic review [155]. 

### 2.10. Macrophage Accumulation and Foreign Body or Giant Cell Formation

Macrophage responses to implanted materials, tendon grafts, or surgical sutures represent an important but often underappreciated aspect of tendon immunobiology. When macrophages encounter materials that exceed their phagocytic capacity—due to particle size, surface chemistry, or persistence—they may undergo cell–cell fusion, forming multinucleated foreign body giant cells (FBGCs). This phenomenon represents a classical foreign body reaction (FBR) and has been documented in tendon tissue, including the Achilles tendon [156,157,158]. FBGCs arise exclusively under pathological conditions and are generated through the fusion of differentiated macrophages [159]. They may contain dozens to over 200 nuclei, forming a syncytial structure in which nuclei cluster centrally or peripherally in a ring-like arrangement [160]. There are two major morphological variants; the first one is foreign body-type giant cells that are typically induced by persistent biomaterials or large particles, which is most relevant to tendon repair and biomaterial implantation and the second is the Langerhans-type giant cells that are more commonly associated with chronic infections or autoimmune processes [160]. In biomaterial-associated FBR, IL-4 and IL-13 serve as key cytokines that drive macrophage fusion. Both induce the expression of fusogenic proteins such as DC-specific transmembrane protein (DC-STAMP) and E-cadherin, mediated through STAT6 signaling [161]. While FBGC formation is traditionally viewed as detrimental, the macrophage fusion response often indicates a sustained attempt to isolate or degrade the foreign material, accompanied by chronic inflammation and fibrosis [162].

Recent work in tendon tissue engineering has increasingly focused on modulating macrophage–biomaterial interactions to minimize FBR and promote regenerative healing. A particularly promising approach is immunomodulatory scaffold design. Dong et al. [163] investigated a polycaprolactone/silk fibroin fiber scaffold functionalized with ECM derived from MSCs. This material reduced pro-inflammatory cytokine secretion, diminished FBGC formation in vivo, and critically enhanced M2 macrophage polarization in a rat Achilles tendon healing model. The shift toward a regulatory macrophage phenotype correlated with reduced adhesions and improved tissue migration, highlighting the potential of immuno-instructive biomaterials in tendon regeneration [163].

Although rare, macrophage-rich lesions may also arise in the form of giant cell tumors of the tendon sheath, a benign neoplasia originating from the tendosynovium. Their pathogenesis is driven by the CSF1-gene rearrangements in neoplastic synoviocytes, which overproduce CSF1 and thereby, recruit large numbers of non-neoplastic macrophages expressing the CSF1 receptor [164]. While typically found in the upper extremities, a recent case report described an unusual presentation near the calcaneus, possibly arising from a tendon sheath or the retrocalcaneal bursa in proximity to the Achilles tendon [165]. These lesions underscore the broader range of macrophage-driven pathologies that can arise in tendon-adjacent tissues.

## 3. Conclusions and Future Direction

Macrophages play a vital and dynamic role in the immunobiology of tendon including the Achilles tendon by coordinating inflammation, repair and regeneration. Their functional plasticity, i.e., context-dependent switching between pro-inflammatory (M1) and anti-inflammatory or reparative (M2) subtypes determines tendon healing outcomes such as regeneration, tendinopathy, delayed healing, fibrosis and scarring or HO. Understanding the balance between macrophages polarization is a key determinant for guiding effective therapeutic strategies.

Future research supported by single-cell omics technologies should focus on macrophage polarization heterogeneity across all tendon compartments (epi-/peri-/endotenon, subfascicular regions), including the enthesis, midsubstance, and myotendinous junction with precise time- and spatial-resolved analyses. Specialized tendon-specific macrophages such as tenophages warrant dedicated investigation.

Moreover, the paracrine actions of macrophage-derived EVs and their influence on TDSCs represent promising yet underexplored therapeutic targets. Advanced models, including hToC systems and coculture approaches, now offer valuable opportunities to comprehensively study macrophage–tenocyte/tendon tissue interactions, simulate physiological or pathological healing environments, and test targeted immunomodulatory interventions.

Early evaluation of macrophage–biomaterial interactions during graft or scaffold development will remain essential to optimize immunomodulatory and regenerative outcomes. Furthermore, PRP therapy provides a clinically relevant approach to modulate macrophage polarization: LP-PRP generally promotes M2 anti-inflammatory and reparative phenotypes, whereas LR-PRP favors M1-mediated early inflammatory responses. Integrating PRP-based strategies with a deeper understanding of macrophage dynamics may enhance tendon repair and improve regenerative outcomes.

## Figures and Tables

**Table 1:** Macrophages polarity in tendon.

	M1	M2	References
**Released factors**	pro-inflammatory cytokines, e.g., TNFα, IL-1β, IL-6, IFNγ, and iNOS	anti-inflammatory cytokines: IL-10, IL-13, TGF-β, PDGF, IL-1 receptor antagonist, VEGF, arginase, IGF-1	[98,99,101,102,103]
**Markers**	CD80, CD86, CD64, CD16	CD206, CD163	[55]
**Properties**	pro-inflammatory: crucial in early stages of inflammation, M1↑: delayed healing	anti-inflammatory: crucial in late stages of inflammation and guide healing, M2↑: scarring, adhesion formation	[64,109,110,118,119]
**Mediators inducing phenotypic shift**	low mechanical strain (3%), low intensity pulsed ultrasound (LIPUS), leukocyte-rich platelet-rich plasma (LR-PRP)	moderate mechanical strain (6%), low intensity LIPUS, mesenchymal stem cells/derived exosomes, leukocyte-poor platelet-rich plasma (LP-PRP)	[11,114,125,145,146]

## Data Availability

No new data were created or analysed in this study. Data sharing is not applicable to this article.

## Abbreviations

The following abbreviations are used in this manuscript:

αSMA	alpha smooth muscle actin
Arg	Arginin
BMP	bone morphogenetic protein
CD	cluster of differentiation
CSF	colony stimulating factor
CX3CL1	fractalkine receptor ligand = C-X3-C motif chemokine receptor ligand
CX3CR1	fractalkine receptor = C-X3-C motif chemokine receptor
DAMP	damage-associated molecular pattern
DC-STAMP	dendritic cell-specific transmembrane protein
ECM	extracellular matrix
EREG	epiregulin
EV	extracellular vesicle
FBGC	foreign body giant cell
FBR	foreign body reaction
FOLR2^+^	folate receptor beta
GM-CSF	granulocyte/monocyte colony stimulating factor
HO	heterotopic ossification
HSP	heat shock protein
hToC	human Tendon-on-a-Chip
IFN	interferon
IGF-1	insulin-like growth factor 1
IKK	IκB kinase
IL	interleukin
iNOS	inducible nitric oxide synthase
JAK	janus kinase
LED LPS	light emitting diode lipopolysaccharide
LP-PRP	leukocyte-poor platelet-rich plasma
LR-PRP	leukocyte-rich platelet-rich plasma
M	Musculus
MCP-1	monocyte chemoattractant protein-1
M-CSF	monocyte colony stimulating factor
MMP	matrix metalloproteinase
MSC	mesenchymal stem cell
NF-κB	nuclear factor kappa B
NLRP3	NLR family pyrin domain containing 3
NO	nitric oxide
NT-3	neurotrophin 3
PAMP	pathogen-associated molecular pattern
PDGF	platelet derived growth factor
PGE2	prostaglandin E2
PRP	platelet-rich plasma
PRR	pattern recognition receptor
PTP1B	protein tyrosine phosphatase 1B
SM-CSF	granulocyte/monocytes colony stimulating factor
STAT	signal transducer and activator of transcription
TDSC	tendon-derived stem and progenitor cell
TGF	transforming growth factor
TH2	helper Type 2 helper T-lymphocytes
TIMP	tissue inhibitors of metalloproteinases
TNF	tumor necrosis factor
Treg	regulatory T cells
VEGF	vascular endothelial growth factor
